# Accessible Multimodal Tool Support for Brainstorming Meetings

**DOI:** 10.1007/978-3-030-58805-2_2

**Published:** 2020-08-12

**Authors:** Reinhard Koutny, Sebastian Günther, Naina Dhingra, Andreas Kunz, Klaus Miesenberger, Max Mühlhäuser

**Affiliations:** 8 Institute Integriert Studieren, JKU Linz, Linz, Austria; 9 Jack Baskin School of Engineering, UC Santa Cruz, Santa Cruz, CA USA; 10 Dipartimento di Meccanica, Politecnico di Milano, Milan, Italy; 11 Support Centre for Students with Special Needs, Masaryk University Brno, Brno, Czech Republic; 12 Institut Integriert Studieren, Johannes Kepler University, Linz, Austria; 13 Technische Universität Darmstadt, Darmstadt, Germany; 14 Innovation Center Virtual Reality, ETH Zurich, Zurich, Switzerland

**Keywords:** Blind and visually impaired people, Brainstorming, Business meeting, Non verbal communication, 2D haptic output device

## Abstract

In recent years, assistive technology and digital accessibility for blind and visually impaired people (BVIP) has been significantly improved. Yet, group discussions, especially in a business context, are still challenging as non-verbal communication (NVC) is often depicted on digital whiteboards, including deictic gestures paired with visual artifacts. However, as NVC heavily relies on the visual perception, whichrepresents a large amount of detail, an adaptive approach is required that identifies the most relevant information for BVIP. Additionally, visual artifacts usually rely on spatial properties such as position, orientation, and dimensions to convey essential information such as hierarchy, cohesion, and importance that is often not accessible to the BVIP. In this paper, we investigate the requirements of BVIP during brainstorming sessions and, based on our findings, provide an accessible multimodal tool that uses non-verbal and spatial cues as an additional layer of information. Further, we contribute by presenting a set of input and output modalities that encode and decode information with respect to the individual demands of BVIP and the requirements of different use cases.

## Introduction

In recent years, the inclusion of persons with visual impairment or blindness has made significant strides towards an accessible society. Digital accessibility, in particular websites and applications found on stationary and mobile devices, has improved tremendously making the consumption of digital information, remote communication and participation over the internet easier than ever before. In contrast, communication during in-situ meetings still heavily relies on non-verbal communication (NVC), which is hardly accessible to blind and visually impaired persons (BVIP) in most situations. In this context, sighted people do not only use their regular means of NVC, such as posture, gaze, facial expressions, or body language [22], but also make use of deictic gestures to refer to visual artifacts or other persons in the room during conversations [11]. While deictic gestures use arm and index finger for referencing points of interest [3, 29] and are learned from childhood on [4, 6], this form of NVC is not self-contained and requires knowledge about where a speaker is pointing at as well as the information which person or artifact a person refers to [1, 7]. Hence, spatial aspects, in particular the location of people and artifacts, play an important role. These spatial aspects are also often inaccessible to BVIP.

Thus, unrestricted participation in group conversations, which additionally make use of visual artifacts like cards or sketches on whiteboards or digital presentations, is still found to be a huge challenge for BVIP. Therefore, a good part of group conversations in a business context is inaccessible for BVIP. Also, tool support for brainstorming meetings has not adequately met the needs of BVIP. In particular, offering means to include a broad variety of user interaction approaches and devices to cater for a wide spectrum of users and use cases as well as considering accessibility has been a shortcoming in this area to date.

In order to address this shortcoming, we propose and implement dynamic tool support for BVIP during business meetings as part of the MAPVI project [13]^1^. Thereby, we allow BVIP to fully participate by providing necessary non-verbal and spatial information as well as providing an accessible user interface to actively contribute on whiteboards. As proof-of-concept, we implement tool support in the form of a Metaplan application used during a brainstorming meeting with an ongoing group conversation.

In this paper, we investigate the requirements of BVIP during brainstorming meetings and present an accessible multimodal tool that utilizes non-verbal and spatial cues as an additional layer of information. We further contribute by presenting a set of in- and output modalities that encode and decode this information with regards to the individual demands of BVIP and requirements of the different use-cases.

The paper is structured as follows: Sect. 2 discusses related work. Section 3 describes the methodology. Section 4 explains the conceptual solution. Section 5 outlines the architecture. Section 6 gives an overview of user-interaction methods possible with this type of approach. Finally, Sect. 7 concludes the paper and gives an outlook on future work.

## State of the Art

Research investigated a large body of interactive systems which allow for body-based [22] or gesture interaction [8, 35], as well as interfaces to allow intuitive world exploration techniques for BVIP [5, 10, 15, 34]. However, during meeting situations, NVC has a tremendous amount of interaction possibilities that can enrich discussions for sighted persons during meeting situations, BVIP are often not able to fully access them and still rely on spoken information [13]. Further, business meetings nowadays usually make use of additional electronic tool support, such as digital presentations, and documents that can be collaboratively edited. Examples range from sticky notes [16] and mind map tools [21] to dedicated brainstorming and decision support software [12]. While all provide collaboration support, none of them provides specialized accessibility interfaces for BVIP.

To address these limitations, research has designed a variety of tools that allow for multimodal interfaces (e.g., [2, 17, 24, 31]) and to support collaboration between BVIP and sighted people. For example, Thieme et al. [30] investigated how tools can assist during collaborative programming tasks. More recently, Shi et al. [28] presented a design tool for teachers to assist students with visual impairments to create interactive tactile maps. With regards to meetings, Regal et al. [27] presented an approach that uses NFC to map digital contents on tangible cards to support brainstorming meetings. Further, in a predecessor project [19], we investigated shortcomings of brainstorming meetings using mind maps and provided an accessible representation of them as an adaptive tree view for BVIP [25, 26]. While this project already revealed great potential, it also exposed the need to support additional modalities to augment the perception of BVIP by providing further methods to handle NVC cues as well as spatial information within business meetings.

In summary, while there are a number of collaborative brainstorming tools that support BVIP through multimodal interfaces, there still remains a need in terms of business meetings, which helps BVIP to alleviate existing constraints that limit the access to non-verbal communication and spatial information, such as the location of participants, collaborative surfaces, or objects.

## Methodology

User acceptance is one of the main success criteria of every project, tool or framework, therefore we involved users from the beginning of the project by interviews with (2 persons, both legally blind) and questionnaires (9 persons, 3 legally impaired and 6 visually impaired). According to these activities NVC is crucial in conversations, especially if more than two people are involved and that there might be disparity between NVC cues BVIP think are helpful during a first interview and after exposure to additional NVC cues. Observations show that the set of NVC cues perceived to be helpful is a very individual matter as well, pointing out the necessity of a highly customizable stream of information conveying NVC cues. The outcomes of these efforts suggest that there is indeed a strong need for technologies, which can improve and support the perception of spatial aspects of group conversations and non-verbal communication for BVIP and a highly customizable solution would be greatly appreciated, since which parts of non-verbal communication are of most interest to a person and which level of detail is necessary and workable is a very individual matter depending on the person and the situation. According to these interviews and questionnaires, an ontology has been developed to describe the information space that is of relevance in this setting. Based on this foundation, the MAPVI brainstorming tool and its architecture were designed to allow for an accessible, extensible and customizable platform supporting a vast amount of devices and methods for user interaction to cater to an as broad as possible user group.

## Concept

The MAPVI Brainstorming tool is developed to manage and support brainstorming meetings together with BVIP. The process and requirements of this application are derived from meeting sessions inspired by the Metaplan method. Therefore, it relies on different roles of participants, in particular a moderator and other participants, referred to as regular participants. These regular participants can be sighted people as well as BVIP. They can create notes, actively participate in the discussion and make decisions, finding consensus as a group. The moderator, on the other hand, asks questions, elicits and organizes ideas and encourages and directs discussions towards specific topics and subtopics arising during this creative process. However, the moderator does not create content or make decisions.

**Fig. 1.**
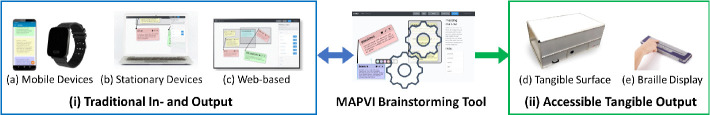
Overview of the MAPVI brainstorming tool that supports (i) traditional in- and output modalities, such as (a) mobile devices, (b) stationary devices and (c) web-based interfaces, as well as (ii) accessible tangible output modalities, such as (d) a magnetic surface with tangible representations and (e) modern braille displays.

These major two roles are also reflected in the brainstorming tool (see Fig. 1). The moderator has his or her own user interface, which allow him or her to organize, place and group notes, created by regular participants. Moderators can also delete notes if requested by participants and agreed upon as a group. This is all done in a web-based application, which shows a virtual whiteboard consisting of notes, groups of notes and relations between notes. Since, whiteboards can contain numerous notes, which can quickly confuse users if presented in one view, multi-view functionality was implemented. This allows multiple views on the same whiteboard, which run simultaneously and show different parts of the whole whiteboard at different zoom levels. The role of regular participants is deliberately restricted to create notes and edit their own notes. This is done via a mobile application, which pushes new notes and changes of existing ones to a server, which is also accessed by the web-based whiteboard application.

## Architecture

### Server-Backend

The brainstorming tool is a client-server application, which consists of two data interfaces: a RESTful API to create, read, update, and delete data (CRUD) and a Web-Socket service for real-time notifications.

**RESTful-API:** Since access to data is not solely be done by one application or framework but multiple different ones, namely, at this stage from a web application and an Android application, a standardized approach is highly beneficial. Therefore, we decided to implement a RESTful-API, which allows for platform-independent, easy access to all necessary data. Via this API, applications of any kind can perform CRUD operations. In particular, data can be accessed concerning meetings in general, users, notes of meetings, groups of notes and relations between notes.

**Authentication:** Typically, this access needs to be restricted to people who are allowed to consume and modify data. To ensure this, we use a token-based authentication mechanism for the RESTful API to ensure the security and privacy of the data entered into the system.

**Web-Sockets:** Because multiple moderator views are possible as well as multiple Android clients can enter and modify data, data gets created, updated and deleted from multiple clients simultaneously. To keep the displayed information updated and consistent, we use Web-Sockets to inform all applications of changes in the data. Applications can register to specific channels, corresponding to data sets. If data gets changed on the server, the server broadcasts this information to appropriate channels and the clients will be informed about these changes.

### Web Interface

The web interface is implemented with Laravel^2^ using Bootstrap and the dynamic part is generated using Konva^3^ JavaScript Library. The web interface displaying the whiteboard view accesses data via the RESTful API and listens to changes via Web-Sockets, for instance, when notes get created by participants using the Android app or get deleted in another instance of the whiteboard view.

### Android App

The Android app accesses data using the RESTful API as well. Changes are communicated via Web-Sockets. Since the role of regular participants is deliberately restricted by the Metaplan technique, the purpose of the Android application, which is only used by regular participants, is very specific and the feature set is comparatively low. Essentially, the Android app allows adding new notes and modify notes that the user-created and it also gives an overview of all notes that were created by an individual user.

## Input and Output Modalities

### General Accessibility of the User Interface

The brainstorming tool supports a broad variety of user interaction methods. Again, the two different roles, moderator and regular participant, play an important role in this context. The moderator user interface, which is, as mentioned before, a web interface, can be operated using a mouse and keyboard, but it can also be operated using a touchscreen. Therefore, big touch screen devices acting as whiteboards are directly realizable. Also, multiple views on the same whiteboard are possible to show different parts of the whole composition of notes and other items. The selected architecture also allows for a keyboard-only operation, if moderators cannot use mice or touchscreens for whatever reason. Screenreader accessibility for the moderator user interface is possible too, because of the way that data is cached and updated in the browser using Web-Sockets.

### Recognition of NVC

One part of the MAPVI project and this software suite is the inclusion of gesture recognition. So far pointing gestures of participants can be recognized [9]. The brainstorming tool allows these pointing gestures to be fed into the system and shared with BVIP. The architecture of the system allows because of the use of an easily extendable RESTful API the implementation of other NVC cues as well. Facial expressions, eye gestures and other hand gestures are possible in the future. This information can be later offered to BVIP.

### Gesture-Based User Interface for BVIP

Additionally, gesture based user interfaces tailored for BVIP are supported, in particular, user interfaces considering spatial aspects of meetings. A mobile application using smartphones in combination with a smartwatch, which is currently under development, can be used to retrieve information about artifacts or participants by pointing in the general direction of information sources using the smartphone or smartwatch as virtual index finger, once this is finished. This will enable BVIP to easier select pieces of information, without cognitively overloading them and shows great potential to improve their spatial perception of the environment including the location of people and artifacts.

### Tangible Representation of the Information

Tangibles provide a haptic experience and are able to map the content of individual items of the digital whiteboard to a physical surface and are distinguishable for BVIP by different shapes and tactile characteristics [14, 18]. Further, they can maintain the position and clustering of the digital cards analogously. Hereby, it is important that the tangibles consistently adapt to the spatial information correctly, which requires an additional mechanism that can move them on the physical surface of the table, e.g., through embedded motors (e.g., [23]) or external mechanical support (e.g., [20]). As tangibles with embedded functionality may increase complexity and are expensive, external mechanisms that can move custom 3D-printed objects are reasonable. For example, electromagnetic arrays (e.g., [32, 33] or a magnetic gripper arm below the surface can precisely address tangibles with magnetic foundations individually and move each object according to the layout of the virtual whiteboard.

We implemented our prototype with a magnetic gripper arm to relocate tangibles to represent the digital content as cards. The spatial information of each card is maintained in real-time to make them physically accessible for BVIP and updates modifications of the spatial distribution of the cards dynamically. A top mounted camera detects the position of each tangible and recognizes changes by the user which triggers update commands to the tool. If a tangible is moved accidentally, the device can return it to its original location to keep the consistency. If a user touches a tangible with the index finger, the linked content of the virtual card of the brainstorming tool is readout.

## Conclusion and Further Work

In this paper, we presented design considerations based on user involvement of members of the target group of BVIP and a proof-of-concept implementation of an accessible multimodal brainstorming tool for business meetings. A careful architectural design and selection of technology have ensured accessibility, extensibility, and customizability of this approach for brainstorming tool support. Multi-model user interface approaches including the tactile representation of information, gesture recognition or gesture-based user interfaces allow BVIP people to gain access to the vast information space of non-verbal communication and show great potential in helping them to participate in group conversations in a business context to the full extent. As the next step, existing user interface approaches will be refined and extended in close cooperation with people of the target group. In particular, gesture-based as well as tactile user interfaces will be investigated in more detail to explore how and to which degree they can help to deal with the immense amount of spatial and non-verbal information that gets generated during group conversations and prevent cognitive overloading blind and visually impaired people. Evaluations with the target group will be undertaken to study the benefits of this approach and reveal areas for improvements.

## Footnotes

1 The MAPVI project including this publication was founded by the Austrian Science Fund (FWF): I 3741-N31.

2 Laravel - PHP web framework: https://laravel.com.

3 Konva - Javascript 2D Canvas Library: https://konvajs.org.
